# Intractable chylous leak after radical esophagectomy treated with radiotherapy

**DOI:** 10.1186/s13019-023-02419-7

**Published:** 2023-11-14

**Authors:** Seha Ahn, Heejin Lee, Joon Kyu Kang, In Sub Kim, Youngkyu Moon, Jung Suk Choi, Yoo Dong Won, Seong Cheol Jeong, Si Young Choi

**Affiliations:** 1 Department of Thoracic and Cardiovascular Surgery, Eunpyeong St. Mary’s Hospital, College of Medicine, The Catholic University of Korea, 1021, Tongil-ro, Eunpyeong-gu, Seoul, 03312 Republic of Korea; 2 Department of Radiology, College of Medicine, Uijeongbu St. Mary’s Hospital, The Catholic University of Korea, Uijeongbu, Republic of Korea; 3 Department of Thoracic and Cardiovascular Surgery, College of Medicine, Uijeongbu St. Mary’s Hospital, The Catholic University of Korea, Uijeongbu, Republic of Korea

**Keywords:** Chylous leak, Radical esophagectomy, Radiotherapy

## Abstract

Postoperative chylous leak after esophagectomy is a rare but potentially life-threatening complication that results in hypovolemia, electrolyte imbalance, malnutrition, and immunologic deficiency. However, the management of postoperative chylous leak remains controversial. Following a diagnosis of esophageal cancer, a 64-year-old man was treated by video-assisted thoracoscopic esophagectomy, laparoscopic gastric tube formation, prophylactically thoracic duct ligation, and reconstruction with esophagogastrostomy at the neck level. Massive postoperative drainage from the thorax and abdomen did not initially meet the diagnostic criteria for chylothorax, which was ultimately diagnosed 3 weeks after the operation. Despite various treatments including total parenteral nutrition, octreotide and midodrine, reoperation (thoracic duct ligation and mechanical pleurodesis), and thoracic duct embolization, the chylous leak persisted. Finally, low-dose radiation therapy was administered with a daily dose of 2 Gy and completed at a total dose of 14 Gy. After this, the amount of pleural effusion gradually decreased over 2 weeks, and the last drainage tube was removed. The patient was alive and well at 60 months postoperatively. Herein, we describe a patient with intractable chylous leak after esophagectomy, which persisted despite conservative treatment, thoracic duct ligation, and embolization, but was finally successfully treated with radiotherapy.

## Introduction

Postoperative chylous leak is a rare but potentially life-threatening complication after thoracic surgery, particularly after esophageal surgery. It is caused by damage to the main thoracic duct or its tributaries, which are anatomically closely related to the esophagus [1]. Persistent chylous leak results in hypovolemia, electrolyte imbalance, malnutrition, and immunologic deficiency [2]. However, an evidence base for the optimal management of postoperative chylous leak after esophagectomy is lacking, which may be associated with the low incidence [3].

We present a 64-year-old man who was treated successfully with low-dose radiotherapy (RT) for intractable chylous leakage after esophageal surgery that had failed various treatments including conservative treatment and thoracic duct ligation and embolization.

## Patient presentation

A 64-year-old man with a history of diabetes mellitus and alcoholic hepatitis was diagnosed with early stage esophageal cancer after a regular medical examination that included an upper gastrointestinal endoscopy. Further diagnostic imaging revealed a primary tumor, 23 cm from the incisor, confined to the esophagus without regional or distant metastases. The patient underwent video-assisted thoracoscopic (VATS) esophagectomy with mediastinal lymph node dissection, laparoscopic gastric tube formation, prophylactically thoracic duct ligation, and reconstruction with esophagogastrostomy at the neck level. The surgery was uneventful, and a right chest tube and abdominal Jackson-Pratt (JP) drain were left for drainage.

**Fig. 1:**
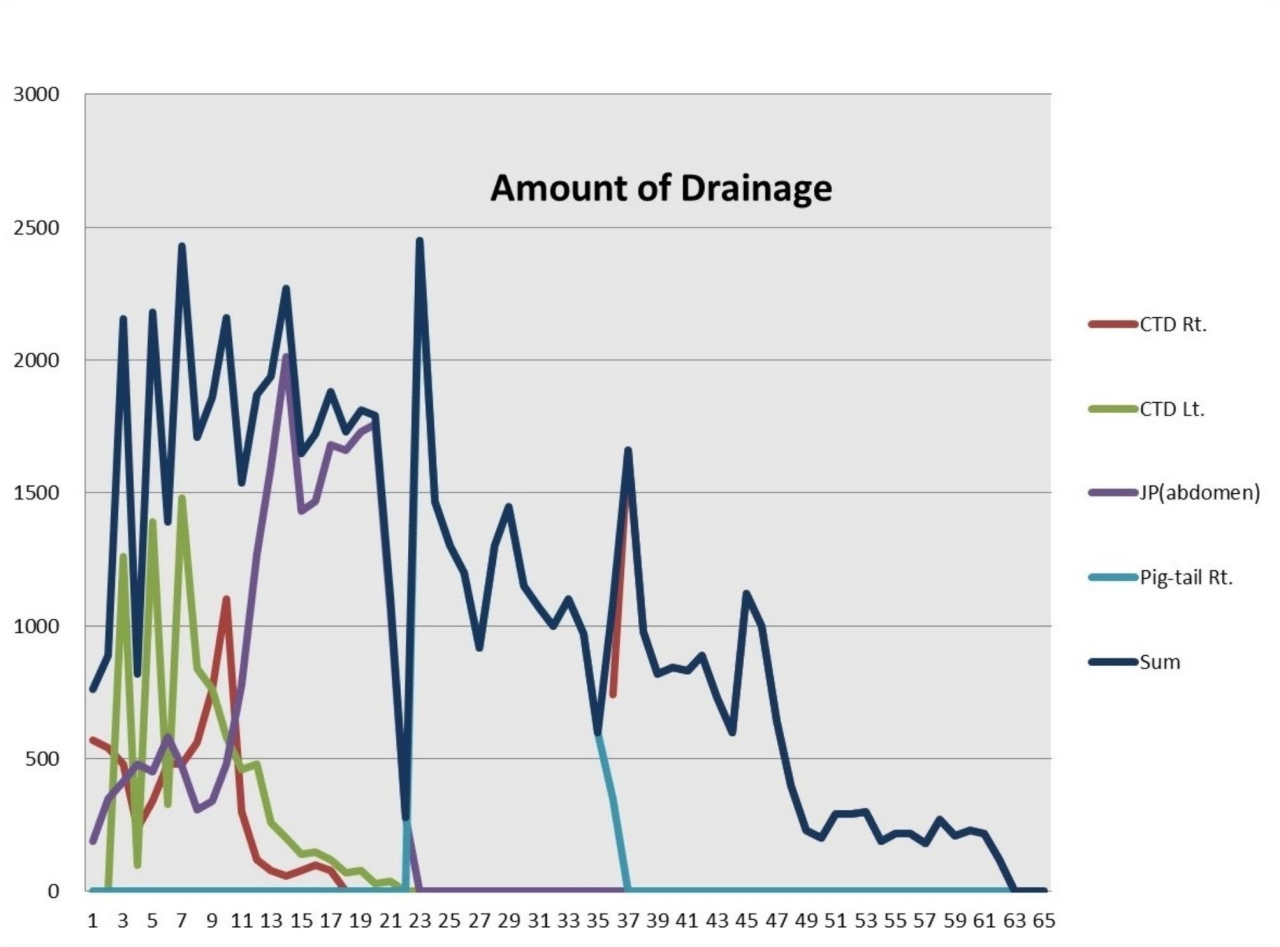
Total fluid output and output volumes from the right chest tube drainage (CTD), left CTD, abdominal Jackson-Pratt drain, and right thoracic pigtail catheter during the postoperative period

On the postoperative day (POD) 2, a chest radiograph revealed a massive left pleural effusion. A chest tube was inserted in the left thoracic cavity and about 1200 mL of effusion was drained. As the effusion was a clear yellow fluid, a wait-and-see approach was taken, but large volumes of pleural and abdominal drainage persisted during the early hospital course. However, the triglyceride concentrations in the drainage fluid were < 110 mg/dL, and the diagnosis of chylothorax was delayed. The right chest tube was removed on POD 17 followed by the left chest tube and the abdominal JP drain on POD 21. Figure 1 shows the total drainage and the drainage amounts from the left and right chest tube drainages (CTD), abdominal JP drain, and a pigtail catheter in the right thoracic cavity during the hospitalization period.

Upper endoscopy on POD 6 showed suspected leakage at the anastomotic site, but esophagography on POD 19 showed no leakage, and the patient began to drink water on POD 20. Then, on POD 22, a chest radiograph revealed a large amount of right pleural effusion, and the pigtail catheter was inserted in the right thorax. The triglyceride content of the fluid was 262 mg/dL, meeting the criterion for chylothorax (> 110 mg/dL). Lymphangiography was performed on the same day.

**Fig. 2:**
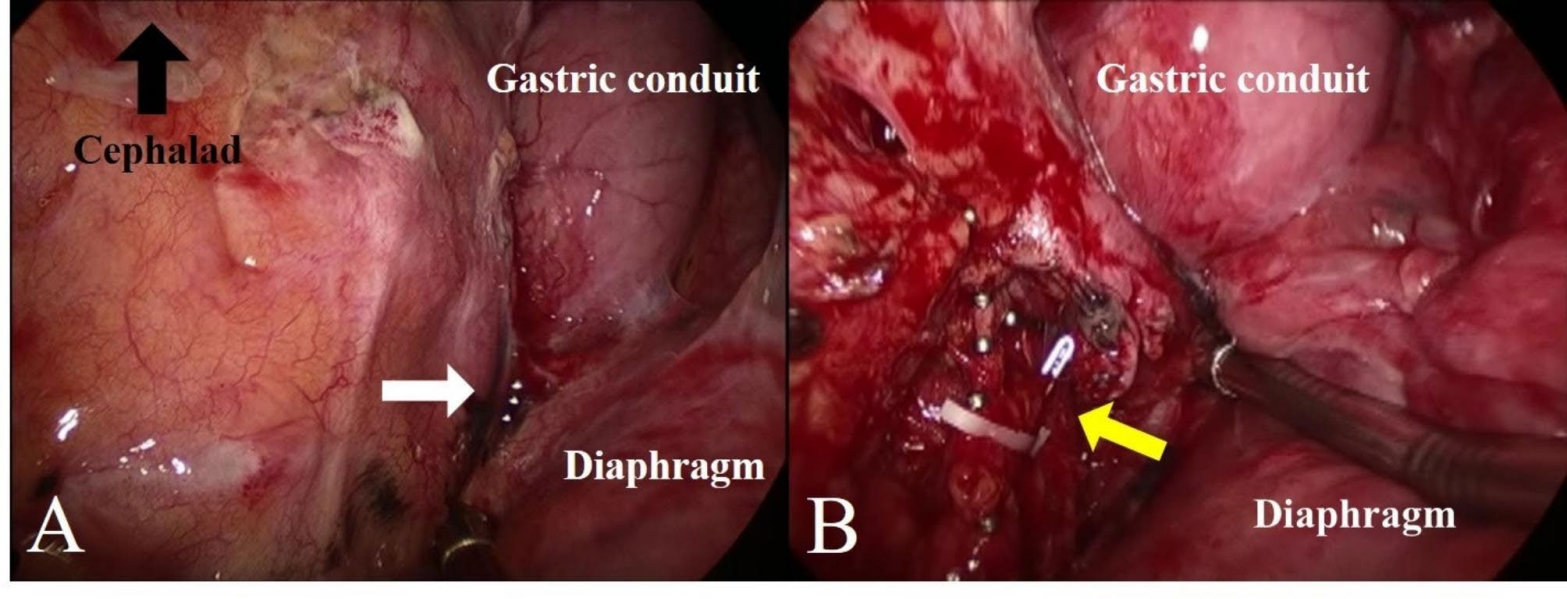
Intraoperative findings: (**A**) visible chylous leakage at the diaphragm (white arrow); (**B**) the thoracic duct after ligation (yellow arrow)

**Fig. 3:**
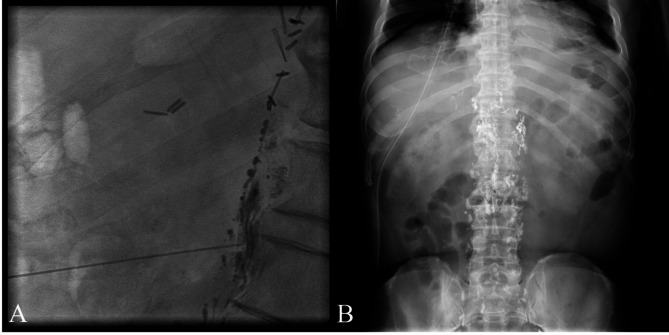
Thoracic duct embolization

After the diagnosis of chylothorax, conservative management began with oral midodrine on POD 23 and intravenous octreotide acetate infusion on POD 25, but the chylous leak was not controlled. The patient was kept nil per os (NPO), and total parenteral nutrition (TPN) was administered through a central venous catheter until POD 34. After several sessions of thorough consultation with the patient and his family, the patient was scheduled for VATS thoracic duct ligation and mechanical pleurodesis on POD 35 (Fig. 2B), during which a chylous leak was identified at the diaphragm (Fig. 2A). Even after the operation, the chylous leak persisted, and a second lymphangiography was performed on POD 40, followed by attempts at thoracic duct embolization on POD 41 and 42, but the effusion did not decrease (Fig. 3).

Finally, we decided on a trial of radiotherapy, and informed consent was obtained from the patient. The clinical target volume encompassed the entire lymph node station, and a daily dose of 2 Gy was administered from POD 43 to POD 50 (14 Gy in 7 fractions). The patient had no acute adverse effects during RT, and the volume of pleural effusion gradually decreased. The right thoracic pigtail catheter was finally removed on POD 64, and the patient was discharged on POD 89. The patient was alive and well at 60 months postoperatively and no late toxicity related to RT was observed.

## Discussion

This case demonstrated that an intractable postoperative chylous leak can be treated successfully with low-dose RT where many other strategies for chylothorax have failed. The most common and initial clinical manifestation of postoperative chylothorax is excessive fluid output or drainage of cream-colored liquid from the chest tube and/or abdominal JP drain [4]. The average daily volume of postoperative pleural and abdominal drainage in our patient was approximately 1670 mL (range 280 to 2450 mL) before the diagnosis of chylothorax on POD 22, but the fluid was not cream-colored, and the average triglycerides were < 110 mg/dL. It is possible that the late diagnosis of chylothorax was related to the patient’s NPO status following esophageal surgery, as our institutional policy is to keep the patient NPO for a week and then perform upper endoscopy or esophagography to confirm a patent cervical esophagogastric anastomosis.

Because the incidence of chylothorax after esophagectomy is low and most studies are retrospective, consensus on the optimal management of postoperative chylous leak is lacking. Power and his colleagues have reported a systematic review and meta-analysis of 25 studies, seeking to identify the optimal approach for chylous leaks following esophageal resection [3]. Our case demonstrates that various approaches including conservative treatment (TPN, octreotide, and midodrine), reoperation (thoracic duct ligation and pleurodesis), lymphangiography, and thoracic duct embolization, and low-dose RT can be attempted to treat the postoperative chylothorax. In our case, after 13 days of conservative treatments following the diagnosis of chylothorax, including oral midodrine, intravenous octreotide acetate, and NPO with TPN, the chylous leakage did not subside. Miao and his colleagues reported that in 11 of 34 patients (32%), conservative treatment (TPN, no enteral intake, and octreotide) failed to successfully stop chylous leak [5]. Surgical repair including thoracic duct ligation (TDL) using VATS is the next step to resolve chylothorax when conservative management has failed [6]. In our patient, even after VATS thoracic duct ligation and pleurodesis, the daily volume of pleural effusion was approximately 1027 mL (range 820 to 1660 mL). Five days after VATS thoracic duct ligation, lymphangiography and thoracic duct embolization were performed twice, but no improvement was seen. Laparoscopic ligation of the cisterna chyli may have been another therapeutic option for our patient, but we did not attempt it because of the complex anatomy of his lymphatic system [7]. Finally, low-dose RT was attempted for the treatment of massive chylous leakage. We treated the clinical target volume, which covered the entire lymph node station. The treatment involved delivering a daily dose of 2 Gy from POD 43 to POD 50, resulting in a cumulative dose of 14 Gy administered over 7 fractions. Although successful treatment of chylothorax with RT has been reported, it has not been widely practiced for the treatment of massive chylous leakage that has not responded to conservative strategies [8–11]. In 2013, Sziklavari and his colleagues investigated the efficacy and outcome of RT for postoperative chylothorax and concluded that RT could be an alternative to repeat major thoracic surgery and be a first choice in postoperative chylothorax treatment [10]. The exact mechanism by which RT stops chylous leakage remains uncertain [11]. Based on findings from previous literature, it is suggested that low-dose radiation primarily influences the modulation of inflammatory reactions, leading to changes in endothelial cell adhesion properties and cellular functions within the lymphatic vessel [9, 11]. Our patient had no acute side effects during and after RT, and the amount of pleural effusion gradually decreased. The last drainage tube was finally removed, and the patient was discharged without further complication.

## Conclusion

We successfully treated a patient with intractable chylous leak after esophagectomy with RT, with no complication, after trials of conservative treatment, thoracic duct ligation, and embolization.

## Data Availability

The data underlying this article will be shared on reasonable request to the corresponding author.

## Abbreviations

RT: Radiotherapy
VATS: Video-assisted thoracoscopic surgery
JP: Jackson-Pratt
POD: Postoperative day
CTD: chest tube drainage
NPO: Nil per os
TPN: Total parenteral nutrition

## References

[1] Cerfolio RJ, Allen MS, Deschamps C, Trastek VF, Pairolero PC (1996). Postoperative chylothorax. J Thorac Cardiovasc Surg.

[2] Machleder HI, Paulus H (1978). Clinical and immunological alterations observed in patients undergoing long-term thoracic duct drainage. Surgery.

[3] Power R, Smyth P, Donlon NE, Nugent T, Donohoe CL, Reynolds JV. Management of chyle leaks following esophageal resection: a systematic review. Dis Esophagus. 2021;34(11). 10.1093/dote/doab012 PMC8597908 33723611

[4] Fujita T, Sato T, Sato K, Hirano Y, Fujiwara H, Daiko H (2019). Clinical manifestation, risk factors and managements for postoperative chylothorax after thoracic esophagectomy. J Thorac Dis.

[5] Miao L, Zhang Y, Hu H, Ma L, Shun Y, Xiang J (2015). Incidence and management of chylothorax after esophagectomy. Thorac Cancer.

[6] Bender B, Murthy V, Chamberlain RS (2016). The changing management of chylothorax in the modern era. Eur J Cardiothorac Surg.

[7] Diaz-Gutierrez I, Rao MV, Andrade RS (2018). Laparoscopic ligation of cisterna chyli for refractory chylothorax: a case series and review of the literature. J Thorac Cardiovasc Surg.

[8] Neu B, Gauss G, Haase W, Dentz J, Husfeldt KJ (2000). Radiotherapy of lymphatic fistula and lymphocele. Strahlenther Onkol.

[9] Mayer R, Sminia P, McBride WH, Stranzl H, Prettenhofer U, Fruhwirth J (2005). Lymphatic fistulas: obliteration by low-dose radiotherapy. Strahlenther Onkol.

[10] Sziklavari Z, Allgäuer M, Hübner G, Neu R, Ried M, Grosser C (2013). Radiotherapy in the treatment of postoperative chylothorax. J Cardiothorac Surg.

[11] Kim SW, Kim JH (2017). Low-dose radiation therapy for massive chylous leakage after subtotal gastrectomy. Radiat Oncol J.

